# Disorders of Sex Development—Novel Regulators, Impacts on Fertility, and Options for Fertility Preservation

**DOI:** 10.3390/ijms21072282

**Published:** 2020-03-26

**Authors:** Nathalia Lisboa Gomes, Tarini Chetty, Anne Jorgensen, Rod T Mitchell

**Affiliations:** 1 Unidade de Endocrinologia do Desenvolvimento, Laboratório de Hormônios e Genética Molecular (LIM/42) da Disciplina de Endocrinologia e Metabologia do Hospital das Clínicas da Faculdade de Medicina, Universidade de São Paulo, 05403 900 São Paulo, Brazil; nathalialisboa.endocrino@gmail.com; 2 Serviço de Endocrinologia da Santa Casa de Belo Horizonte, Av. Francisco Sales, 1111, 30150-221 Belo Horizonte, Minas Gerais, Brazil; 3 Department of Diabetes and Endocrinology, Royal Hospital for Sick Children, 9 Sciennes Road, Edinburgh EH9 1LF, UK; tarini.chetty@nhslothian.scot.nhs.uk; 4 Department of Growth and Reproduction, Rigshospitalet, University of Copenhagen, Blegdamsvej 9, 2100 Copenhagen, Denmark; anne.joergensen.02@regionh.dk; 5 International Research and Research Training Centre in Endocrine Disruption of Male Reproduction and Child Health (EDMaRC), Blegdamsvej 9, 2100 Copenhagen, Denmark; 6 Medical Research Council (MRC) Centre for Reproductive Health, The University of Edinburgh, The Queen’s Medical Research Institute, 47 Little France Crescent, Edinburgh EH16 4TJ, UK

**Keywords:** disorder of sex development, fertility, fertility preservation, gonads, testis, ovary, sex determination

## Abstract

Disorders (or differences) of sex development (DSD) are a heterogeneous group of congenital conditions with variations in chromosomal, gonadal, or anatomical sex. Impaired gonadal development is central to the pathogenesis of the majority of DSDs and therefore a clear understanding of gonadal development is essential to comprehend the impacts of these disorders on the individual, including impacts on future fertility. Gonadal development was traditionally considered to involve a primary ‘male’ pathway leading to testicular development as a result of expression of a small number of key testis-determining genes. However, it is increasingly recognized that there are several gene networks involved in the development of the bipotential gonad towards either a testicular or ovarian fate. This includes genes that act antagonistically to regulate gonadal development. This review will highlight some of the novel regulators of gonadal development and how the identification of these has enhanced understanding of gonadal development and the pathogenesis of DSD. We will also describe the impact of DSDs on fertility and options for fertility preservation in this context.

## 1. Gonadal and Germ Cell Development

Genetic sex is determined from the point of conception. In contrast, the developing gonad remains bipotential until approximately six weeks post-conception, as until this point it can develop into an ovary or a testis. Development of the gonad can be divided into two key phases, an initial phase with formation of the bipotential gonad and a second phase with differentiation towards either testicular or ovarian fate.

Bipotential gonads arise at approximately four weeks post-fertilization in the human embryo, as paired structures known as genital ridges formed from a thickening of intermediate mesoderm and proliferation of overlying coelomic epithelium [1]. The gonads emerge from the ventral surface of the cranial mesonephros, which in addition give rise to the adrenal glands [2]. Each mesonephros also contains the mesonephric duct (Wolffian duct) and paramesonephric duct (Mullerian duct), precursors of the male and female reproductive tracts respectively [3,4]. The bipotential gonad is initially exclusively comprised of somatic mesoderm derived cells, which are the precursors of steroid producing testicular Leydig and ovarian theca cells and of the supporting Sertoli cells (in the testis) and granulosa cells (in the ovary). Subsequently, by five weeks post-conception, primordial germ cells migrate from the yolk sac to the urogenital ridge. Several genes including *NR5A1* (also known as *SF1*), *WT1*, *EMX2*, and *LHX9* are required for the formation of the bipotential gonadal ridge in humans [5,6].

Traditionally, differentiation of the bipotential gonad into a testis or ovary was thought to be due solely to the presence or absence of the *SRY* gene on the Y chromosome, with ovarian development occurring in the absence of SRY expression. However, it is increasingly recognized that there are in fact several gene networks involved in the complex process of gonadal differentiation towards either ovarian or testicular fate, some of which act antagonistically on the opposite pathway.

In the XY gonad, at approximately seven weeks post conception, SRY is expressed in Sertoli cell precursors, and can be thought of as the dominant ‘switch’ in promotion of testicular development [7]. SRY acts on SOX9, which reaches a critical threshold to drive positive regulatory loops that maintain high levels of SO9 expression and activity independent of SRY expression [5,8]. SOX9 then initiates differentiation of the supporting cell lineage into Sertoli cell rather than granulosa cell fate. SOX9 may also have a role in repressing genes involved in ovarian development such as *WNT4*, *FOXL2*, and transcription factor *DMRT1* [9,10].

Sertoli cells secrete anti-Mullerian hormone (AMH), which, via bone morhogenic protein (BMP)-like signaling pathways, promotes regression of Mullerian structures in males [11]. In addition, Sertoli cells secrete desert hedgehog (DHH) which induces development of steroid producing fetal Leydig cells that secrete testosterone and INSL3 from approximately eight to nine weeks of development [12]. Testosterone promotes differentiation of the Wolffian duct into epididymis, vas deferens, and seminal vesicles and, along with INSL3, contributes to testicular descent [13]. From around eight weeks post-conception, dihydrotestosterone (DHT), produced primarily by enzymatic conversion of testosterone, acts on the androgen receptor causing virilization of the external genitalia [14].

Ovarian development is an active process involving antagonistic regulatory networks that suppress testis development. Absence of SRY expression prevents SOX9 reaching a critical threshold, and this combined with the expression of factors such as RSPO1, WNT4, and FOXL2 that act to suppress testicular development, results in formation of an ovary [8]. In the XX gonad, low levels of AMH and the absence of testosterone cause Wolffian ducts to involute and Mullerian ducts to develop to form the oviduct, uterus, and upper part of the vagina. Absence of androgens results in the development of external female genitalia.

The presence of germ cells and their capacity to generate gametes is essential for future fertility. During fetal development, primordial germ cells migrate into the developing gonad from approximately five weeks gestation at which point they are termed gonocytes [15]. Gonocytes in both sexes express pluripotency markers and undergo further development towards a spermatogonial or oogonial fate. In males, gonocytes begin to downregulate pluripotency factors early in the second trimester leading to the development of (pre)spermatogonia [16]. Failure of germ cells to transition from gonocyte to spermatogonia in fetal and early postnatal life may result in the development of precursor cells for testicular germ cell tumours [17], whilst loss of germ cells at any stage of development may impact on fertility potential. Spermatogonia represent the germ cell population in the prepubertal testis. At puberty, meiosis is initiated and completion of spermatogenesis results in production of a continuous supply of spermatozoa from a self-renewing pool of spermatogonial stem cells (SSC). In contrast, female germ cells (oogonia) enter meiosis from around 10 gestational weeks and become oocytes [18]. Importantly, germ cell survival and development in both sexes is dependent on unique interactions with the somatic cell populations of the gonad; therefore, failure in the development of both somatic cells and germ cells during gonad development can impact on germ cells and future fertility.

## 2. Disorders of Sex Development

Disorders (or differences) of sex development (DSD) are a heterogeneous group of congenital conditions associated with variations of chromosomal, gonadal, or anatomical sex [19]. This diverse group of conditions most often present in the newborn period with ambiguous genitalia or in adolescence with atypical pubertal development. DSDs, as described in the Chicago consensus statement, can be classified into three broad categories: sex chromosome, 46,XY, and 46,XX DSD [19]. 

Sex chromosome DSD encompasses differences in sex chromosome copy number including Turner and Klinefelter syndromes. Turner syndrome (TS) is characterized by 45,X monosomy, the presence of an abnormal X chromosome, or mosaicism of the 45,X cell line (e.g., 45,X/46,XY). Pure 45,X monosomy is associated with a more severe phenotype of severe short stature, gonadal dysgenesis, and dysmorphic features compared to mosaic genotypes who may enter puberty spontaneously before developing primary ovarian failure, premature loss of oocytes, and infertility. Klinefelter syndrome is characterized by the presence of a 47,XXY cell line and clinical features include tall stature, small testes, gynecomastia, primary gonadal failure, progressive germ cell loss, and infertility [20]. 46,XX/46,XY mosaicism and 46,XX/46,XY chimerism are associated with dysgenetic gonads, infertility, and a highly variable phenotype which may present with ambiguous genitalia at birth [21].

46,XY DSD is usually characterized by ambiguous or female appearance of external genitalia with or without the presence of Mullerian structures as a result of under virilization in utero. They can be further subdivided into three diagnostic categories: problems with gonadal development resulting in gonadal dysgenesis, biosynthetic defects causing impaired production of androgens (testosterone and dihydrotestosterone), or lack of androgen action due to end organ resistance to these hormones. The 46,XY DSDs that result in impaired gonadal development are also associated with abnormalities of germ cell development predisposing to gonadal tumors and infertility, with the relative risk of malignancy dependent on the specific condition [22].

46,XY complete gonadal dysgenesis (CGD), also known as Swyer syndrome, is characterized by failure of testicular development and formation of fibrous streak gonads which lack germ cells. Lack of production of AMH and testosterone results in a completely female appearance of the external genitalia and well developed Mullerian structures [23]. In contrast, partial gonadal dysgenesis (PGD) is characterized by partial testis development and can present with a spectrum of phenotypes with a variable degree of masculinization of the external genitalia and a combination of Wolffian and Mullerian ducts. Rarely, there can be the presence of both ovarian and testicular tissue in one individual, known as 46,XY ovotesticular (OT) DSD. 

Androgen biosynthetic defects high in the steroid pathway such as lipoid congenital adrenal hyperplasia (CAH); caused by an abnormality in the steroidogenic acute regulator (StAR) protein) can result in a similar biochemical picture to that of gonadal dysgenesis with low basal and stimulated testosterone levels and low testosterone precursors. In contrast, biosynthetic blocks further down the steroid pathway result in low testosterone levels with high steroid precursors and include CAH variants such as 17-α hydroxylase/17,20-lyase deficiency, 3-beta-hydroxysteroid dehydrogenase, and 17-beta-hydroxysteroid deficiencies. Disorders of androgen biosynthesis can impair germ cell development resulting in infertility [24].

Dihydrotestosterone is formed by enzymatic reduction of testosterone by 5-α reductase via the classical pathway [25], and by an alternative ‘backdoor pathway’ where 17α-hydoxyprogesterone (17OHP) is converted to DHT bypassing the intermediates of testosterone and androstenedione [14]. Defects in either of these pathways can result in DHT deficiency and incomplete virilization of the external genitalia with normal Wolffian structures. Mutations in *SRD5A2* cause 5-α reductase type 2 deficiency, an autosomal recessive condition that classically presents with a female phenotype at birth and significant virilization without breast development at puberty. Impaired spermatogenesis is common in individuals with 5-α reductase deficiency [26].

46,XY DSD can be caused by defects in androgen action, typically due to dysfunction of the androgen receptor (AR) [27]. DSD due to complete loss of function of the AR is termed Complete Androgen Insensitivity Syndrome (CAIS), whereas mutations that retain some residual function result in Partial Androgen Insensitivity Syndrome (PAIS). CAIS typically presents in adolescence as primary amenorrhea despite normal breast development. Alternatively, CAIS may present in infancy with palpable inguinal masses in an individual with a 46,XY karyotype and a typical female appearance to the external genitalia. The phenotype in PAIS is variable depending on the degree of androgen sensitivity and there may be associated gynecomastia due to the peripheral conversion of testosterone to estradiol. Individuals with inactivating mutations of the LH receptor (Leydig cell hypoplasia) have a variable appearance ranging from a completely female phenotype to a variable degree of virilization, very similar to patients with AIS. However, breast development is not observed. Fertility is impaired in the majority of individuals with disorders of androgen action as a result of germ cell loss and/or failure of spermatogenesis [28].

46,XX DSD is usually characterized by ambiguous or virilized genitalia as a result of fetal exposure to androgen excess and normal development of Mullerian structures and ovaries. Androgens may be derived from the fetal adrenal gland such as in CAH or placental aromatase deficiency, or rarely from exogenous sources such as transplacental passage of androgens from a maternal adrenal or ovarian tumor. Exposure to androgens can impact fertility in 46,XX individuals with DSD despite the presence of normal female reproductive anatomy [24]. Androgen arising from isolated testicular tissue (46,XX testicular DSD) or from a mixed gonad of testicular and ovarian tissue (46,XX ovotesticular DSD) occurs in a rare subgroup of 46,XX DSD. Germ cell development is impaired in these individuals with impacts on fertility [29].

## 3. Novel Regulators and Mechanisms of Gonadal Development 

The gonadal determination process is complex and involves numerous genes that are expressed in a tightly regulated temporo-spatial manner in order to lead not only to gonadal differentiation, but also its maintenance. The majority of the patients with 46,XY gonadal dysgenesis (GD), *SRY* negative 46,XX ovotesticular (OT), and 46,XX testicular (T) DSD remain without a molecular diagnosis, indicating that novel genes, genomic rearrangements, and unknown regulatory regions could be involved in these disorders. 

Recently, novel insights into the pathogenesis of human DSD have resulted from the advent of massively parallel sequencing technologies, bioinformatics and innovative tools for the generation of animal models, such as CRISPR/CAS9. This has increased our understanding of how novel genes, in addition to those previously known to be associated with DSD, are involved. Also, this has identified mechanisms and pathways dysregulated in human DSDs. The identification of the underlying genetic basis for a specific DSD may lead to options for individualized management regarding genetic counseling and novel therapeutic interventions, in addition to assessment and management of fertility.

### 3.1. The SRY/SOX Family: New Concepts in Gene Dosage and Regulation

It is known that presence of the *SRY* gene tips the balance to promote testis development, leading to an up-regulation of *SOX9* expression at around 6 weeks gestation in humans and 10.5 days post-coitum in mice [7,30,31,32]. However, until recently, the mechanism by which *SRY* activates *SOX9* was not understood in detail in humans and was only partially elucidated in mice.

In mice, *Sry* binds synergistically with *Sf1* (encoded by *Nr5a1*) to activate a *Sox9* enhancer, known as Testis Specific Enhancer of Sox9 (TES) and its core sequence known as TESCO [33,34]. Deletion of TESCO or TES reduced *Sox9* expression levels in XY fetal mouse gonads to 60% or 45%, respectively, compared to wild-type testis [34]. However, loss of function of TESCO was insufficient to cause sex reversal, indicating that TESCO is not the only *Sox9* enhancer in mice [34]. In humans, no single variant in this region had been identified [35].

Recently, Gonen and colleagues identified a novel gonadal regulatory element upstream of *Sox9*, enhancer 13 (Enh13), which was shown to be essential in the initiation of mouse testicular differentiation [36]. Deletion of Enh13 led to complete XY male-to-female sex reversal in mice. Importantly, Enh13 is conserved and embedded within a 32.5-kilobase region in humans in which deletions have been associated with XY sex reversal, suggesting that Enh13 may also be critical in humans [36].

Since 2011, copy number variants (CNVs) located within the 2 Mb putative *SOX9* upstream regulatory region, denoted XYSR and RevSex have been increasingly identified by array comparative genomic hybridisation (CGH) or multiplex ligation-dependant probe amplification (MLPA) in several isolated 46,XX and 46,XY DSDs (reviewed in [37]). The refined analysis of these genomic regions has allowed the identification of two putative enhancers 5′ of *SOX9*, named Sex Reversal Enhancer-A (eSR-A) and Sex Reversal Enhancer-B (eSR-B) [38]. Both of these enhancers are activated by SOX9 alone or in combination with NR5A1, but have little activation by SRY, the primary initiator of the testis determination. This experimental data was corroborated by the fact that loss of one copy of those elements in 46,XY DSD patients with either complete or partial gonadal dysgenesis appeared to be sufficient to prevent upregulation or maintenance of SOX9 expression to the levels required to ensure proper testis development. By contrast, a single additional copy of either of these enhancers promotes the expression of SOX9 to a level that is sufficient to override the ovarian program causing testicular or ovotesticular DSD in 46,XX individuals [38].

The identified eSR-A showed 80% sequence conservation with its orthologous mouse enhancer Enh13 [36]. In contrast to the human enhancer, Enh13 demonstrated strong enhancer activity in response to SF1 in combination with SOX9 and also with SRY. Thus, different SOX9 activation mechanisms may be observed between species [38]. 

An additional enhancer, Alternate Long-Distance Initiator (eALDI), was also identified as an SRY-responsive enhancer of SOX9, which is 1.4 kb upstream of human TESCO. This enhancer is significantly activated by co-transfection with SRY+SF1 or SOX9+SF1, but not with SOX9 alone. Therefore, eALDI appears to be the primary enhancer by which SRY and SF1 act to initiate SOX9 expression; SOX9 alone or in combination with SF1 can then upregulate expression via all three enhancers, which may together act on the SOX9 promoter through chromatin looping events. Despite the evidence implicating eALDI as an enhancer of SOX9, eALDI CNVs have not been identified in DSD patients [38]. Also, the exact order in which enhancer activation occurs remains elusive given the limitations of studying early human embryonic development. 

Despite the fact that *SOX9* is the only recognized SRY target gene, other SOX genes can mimic its functions. The SRY-related HMG box 3 gene (*Sox3*), which is structurally very similar to *Sry*, is predominantly expressed in developing neural tissue, with no function during sex determination [39,40]. However, copy number variations (CNVs) identified in *SOX3* regulatory regions have been detected in patients with 46,XX testicular and OT DSD [41,42]. Transgenic mouse models confirmed that ectopic gonadal expression of Sox3 induces testis differentiation by upregulating Sox9 in a similar manner to Sry, suggesting that these genes may be functionally interchangeable in sex determination [42]. 

Additionally, previous data suggested that Sox8 was a contributor to murine testis-determination and maintenance of gonadal function [43,44]. SOX8 is co-expressed with NR5A1 and SOX9 in the early stages of human testis-determination as well as in Sertoli and Leydig cells in adult men [45]. In 2018, two rearrangements involving the *SOX8* locus and one missense heterozygous variant in *SOX8* (c.468G>C; p.Glu156Asp) were identified in 46,XY DSD patients, and other missense variants were associated with male infertility and ovarian insufficiency in females [45]. All these variants showed an altered SOX8 biological activity compared to the wild-type protein.

Despite the fact that no data has been reported on fertility in these patients, a study published in 2019 was the first to link the SOX9 pathway as a possible future target for infertility intervention [46]. The identification of deleterious homozygous variants in the *PPP2R3C* gene (which encodes protein phosphatase two regulatory subunit B″gamma), in four syndromic girls with 46,XY complete gonadal dysgenesis from four unrelated families led to the genetic study of the infertile cases in the affected families and parents who harbored the variants in heterozygous state. All three studied fathers, including one reported as infertile, were found to have teratozoospermia with severe head, acrosomal, and nuclear abnormalities following semen analysis. One mother reported oligomenorrhea and hypomenorrhea with no abnormality in pelvic ultrasonography and another mother had menopause at 44 years of age [46]. 

PPP2R3C encodes B″gamma regulatory subunit of PP2A and SOX9 is a downstream canonical target of PP2A. SOX9 needs to be phosphorylated to SOX9-Phospho to be activated to induce downstream pathways for testicular development. The study showed that the variants in PPP2R3C were predicted to upregulate the catalytic function of PP2A, subsequently increasing the dephosphorylation of phosphorylated SOX9, leading to disruption in testis development [46]. Another previous study showed that PP2A may play an important role in testicular development and in spermatogenesis [47]. The presented data provided evidence on the key role of PPP2R3C protein not only in testicular development, but also as a critical intracellular signaling molecule involved in spermatogenesis, showing that heterozygous defects of this gene are associated with impaired spermatogenesis and male infertility. The authors mention that selective pharmacologic PP2A inhibitors, such as okadaic acid, were shown to promote fertility by increasing sperm motility, velocity, and lateral head amplitude [48,49]. Thus, these findings link specific genetic causes of human DSD with novel therapeutic targets of human infertility.

### 3.2. NR5A1 (SF1) and WT1: Old Genes, New Mechanisms

As an important regulator of adrenal function, the first *NR5A1* homozygous mutations were identified in two patients with primary adrenal insufficiency and 46,XY complete gonadal dysgenesis [50,51]. After the identification of a patient with partial gonadal dysgenesis without adrenal insufficiency [52], an increasing number of heterozygous variants in this gene were identified in patients with a range of 46,XY DSD phenotypes without adrenal insufficiency, including patients with complete and partial GD, isolated hypospadias, and bilateral anorchia [53,54]. Thus, *NR5A1* variants are considered one of the most common causes of 46,XY DSD [55]. In addition, *NR5A1* variants are a recognized cause of isolated male infertility. This includes reports of men with azoospermia or severe oligozoospermia (with and without previous cryptorchidism), some whom also had low testosterone and elevated gonadotropins [56].

However, the precise mechanism by which *NR5A1* action fails and leads to DSD is not fully understood, nor has an explanation been provided for the wide-ranging phenotypes associated with different and, in some cases identical, *NR5A1* variants. This is despite numerous studies attempting to resolve this question by analyzing defective NR5A1 function at steroidogenic target promoter (such as *CYP11A1, CYP17A1, and CYP19A1*) or sex differentiation genes (*AMH* and *INSL3*) [57]. 

A recent analysis of TESCO activation together with NR5A1 nuclear localization showed correlation, at least for most of the studied cases, with the phenotypic severity. This indicates that defective TESCO/SOX9 activation may account for the high phenotypic variability in patients with 46,XY DSD harboring deleterious variants in *NR5A1* [58]. Other studies have suggested that this variability is due to digenic/oligogenic inheritance as rare variants identified in additional DSD genes in several patients with *NR5A1* mutations [59,60,61,62]. 

In 46,XX individuals, *NR5A1* variants were previously only associated with ovarian insufficiency [53,54]. However, in 2016, a single recurrent heterozygous *NR5A1* variant (p.Arg92Trp) was identified in patients with 46,XX testicular and OT DSD by three independent groups [1,63,64]. Subsequently, additional cases have been reported [54], all without evidence of adrenal insufficiency. A different homozygous variant involving the same amino acid (p.Arg92Gln) was identified in two patients with adrenal insufficiency, a 46,XX girl [65] and a 46,XY GD individual [51], while this same variant was identified in a heterozygous state in a 46,XX OT individual without adrenal insufficiency [66]. A third heterozygous *NR5A1* mutation (p.Ala260Val), was recently described in a 46,XX OT DSD individual [67].

The wide phenotypic variability observed among 46,XX and 46,XY DSD individuals, with and without adrenal insufficiency, has been explored using in vitro assays. During normal ovarian development, NR5A1 and β-catenin form a complex that upregulates the *Nr0b1* gene, which is involved in SOX9 repression in 46,XX individuals. The *NR5A1* p.Arg92Trp and p.Ala260Val variants impede the action of the NR5A1/β-catenin complex interaction and thus undo the NR0B1-mediated repression of SOX9 probably leading to a disruption in ovarian determination [1,63,64,67]. In 46,XY individuals, the p.Arg92Trp variant reduced activation of several minimal promoters (AMH, CYP11A1) and enhancers (SOX9, TESCO) involved in testis development, thus explaining the GD phenotype [1]. This variant also exhibited partial loss of DNA binding and transcriptional activity, explaining the adrenal insufficiency phenotype when transmitted in homozygous state [51]. However, it remains unclear why there are 46,XX and 46,XY individuals who are asymptomatic and fertile carriers of these variants [1,51,63].

*WT1* deleterious variants were previously only associated with 46,XY DSD in Denys-Drash and Frasier syndromes. In recent years, the involvement of *WT1* in XX gonadal development has been demonstrated by the description of two deleterious heterozygous variants in two 46,XX patients with premature ovarian insufficiency (POI) [68] without kidney disease, and also in a patient with POI and adult-onset focal segmental glomerulosclerosis [69]. Recently, a de novo frameshift variant of WT1 was identified in a girl with 46,XX testicular DSD, c.1453_1456del, p.(Arg485Glyfs*14). Structural protein remodeling suggests an increased activation of target genes, mainly NR5A1. However, these assumptions remain to be experimentally validated [70]. 

### 3.3. PBX1 and CBX2: Gene Interactions that Promote Testis Development

The TALE homeodomain of Pre-B-Cell Leukemia Transcription Factor 1 (*Pbx1*) is known to play an important role in mouse adrenal and urogenital development [71]. At E14.5, *Pbx1*^−/−^ mice exhibit severely impaired testis development associated with markedly decreased cell proliferation in the genital ridge [71].

In humans, deleterious variants involving *PBX1* were previously associated with congenital anomalies of the kidney and urinary tract, but not with DSD [72,73]. *PBX1* has been implicated in human testicular development after the identification of a single de novo heterozygous variant (p.Arg235Gln) in a 46,XY GD patient with normal kidneys and radiocubital synostosis [74]. This variant is located in a highly conserved TALE homeodomain of the protein, as opposed to previously described variants that are located in the consensus splice site, potentially explaining the different phenotype [72,73]. In vitro studies of cellular location showed that the mutated p.Arg235Gln PBX1 protein was unable to correctly localize into the nucleus and also had an impaired biological activity, as its physical interaction with two proteins known to be involved in testis determination (EMX2 and CBX2) were abolished [74]. This data suggests that specific variants located in the TALE homeodomain of PBX1 are a novel cause of human 46,XY DSD.

*CBX2* isoform 1 is thought to lie upstream of *SRY* gene expression in the human sex development cascade based on the findings in mouse models. *Cbx2* (M33) knockout mice have hypoplastic gonads in both sexes and, in the XY mice, the expression of *Sry* and *Sox9* is reduced [75,76]. Additionally, it has been shown that forced expression of *Sry* or *Sox9* in Cbx2 XY KO mice could rescue their sex reversal, although they present with smaller gonads compared to wild-type mice [76]. This data suggests that Cbx2 regulates *Sry* expression in gonadal development and might also influence gonadal size.

In humans, compound heterozygous *CBX2.1* variants were identified in a 46,XY DSD patient with complete gonadal dygenesis and histologically normal ovaries, resembling the knock-out (KO) XY mice phenotype [77]. Functional studies demonstrated that these variants do not bind to, or adequately regulate the expression of, target genes important for gonadal development, such as *NR5A1* [77].

A more recent in vitro study showed that Cbx2.1 also mediates repression of bivalent ovary determining genes, such as the downstream Wnt signaler Lef1 in mice [78]. In addition, functional analysis revealed that CBX2.1 is upstream of genes contributing to ovarian function including folliculogenesis and steroidogenesis and it also regulates genes associated with POI, such as *POF1B*, *BMP15*, and *HOXA13*, suggesting that *CBX2.1* is essential for gonad formation in both sexes [79].

Another *CBX2* isoform (*CBX2.2*) was implicated in the aetiology of 46,XY GD after the identification of deleterious heterozygous variants in two patients with 46,XY partial GD. These *CBX2.2* variants failed to regulate the expression of genes essential for gonad development, primarily *EMX2* [80]. CBX2 isoforms are a cause of 46,XY GD and to date no information about fertility potential has been reported.

### 3.4. NR2F2: A “Pro-Ovary and Anti-Testis” Gene

The orphan nuclear receptor *NR2F2* gene, which encodes the transcription factor chicken ovalbumin upstream promoter transcription factor 2 (COUP-TF2), has been described as a “pro-ovary and anti-testis” gene following the identification of two frameshift variants in three syndromic 46,XX children, one with ovarian dysgenesis and the other with OT DSD, associated with congenital heart disease and variable somatic anomalies including blepharophimosis-ptosis-epicanthus inversus syndrome (BPES) [81]. A 3 Mb deletion containing the *NR2F2* gene was also described in an individual with 46,XX OT DSD with a similar phenotype [82]. BPES in usually caused by heterozygous loss-of-function *FOXL2* variants with or without ovarian dysgenesis. The precise mechanism by which COUP-TF2 defects leads to 46,XX DSD has not been elucidated. However, at the initiation of ovarian development, FOXL2 and COUP-TF2 appear to be mutually exclusive at the cellular level, with distinct location in the somatic and stromal cells of the fetal ovary, respectively [81]. COUP-TF2 is expressed at the same time as WT1 in early gonadal embryogenesis [83] and it regulates negatively the expression of the pro-testis *Sox9* gene in the osteogenic mesenchyme. Hence, it is hypothesized that testis development in these individuals with *NR2F2* mutations is driven by SOX9 activation via WT1 [82].

In addition, COUP-TF2 is responsible for eliminating the male reproductive tract in female embryos through its activation in the Wolffian duct mesenchyme, independently of the absence of androgens [83]. In 46,XY individuals, COUP-TF2 is expressed in Leydig cells from the adult population and is known to bind to DNA sequences similar to NR5A1 elements. Both of them regulate *Star* [84] and *Insl3* [85] gene expression in Leydig cells. Recently, it was shown that not only is NR5A1 capable of activating *Amhr2* gene expression in Leydig cells, but also COUP-TF2, independently of NR5A1 activation [86]. No *NR2F2* variants havebeen described in 46,XY individuals. However, in mouse models, inactivation of *Nr2f2* during prepubertal stages of male sexual development results in infertility, hypogonadism, and a block in spermatogenesis due to a failure of progenitor Leydig cells to mature and produce sufficient testosterone [86].

### 3.5. FGF and WNT Signaling: Antagonistic Pathways in Gonadal Sex Differentiation

The sex-specific differentiation of gonads is controlled through a combination of signaling factors promoting either male or female differentiation and antagonism of the opposite pathway (reviewed in [87]). Studies in mice have elegantly demonstrated that FGF9 and WNT4 act antagonistically on the female and male promoting pathways, respectively [88]. The loss of either *Fgf9* or *Fgfr2* in XY gonads results in elevated expression of *Wnt4* and subsequently in complete male-to-female sex reversal. However, when the female-promoting factor *Wnt4* is simultaneously ablated, testicular differentiation of the gonad is promoted, suggesting that the primary role of FGF9/FGFR2 signaling is the repression of female-promoting genes [89]. Interestingly, the relationship between these two signaling pathways does not appear to be completely symmetrical since loss of *Fgf9* in XX *Wnt4^−/−^* gonads do not rescue the observed partial female-to-male sex-reversal [89]. 

Consistent with a conserved role for FGF9/FGFR2 signaling in human gonadal development, a heterozygous missense mutation (c.1025G>C, p.Cys342Ser) in the *FGFR2c* gene was associated with complete 46,XY gonadal dysgenesis with a female phenotype [90]. No information about fertility potential was reported for the patient with this specific mutation who was gonadectomized at age 15 years due to bilateral dysgerminoma. Additional antagonistic interactions between testis- and ovary-promoting pathways have been identified in mice. FGFR2c and FOXL2 display such an antagonistic relationship based on the finding of complete sex reversal in XY *Fgfr2c^−/−^* gonads, but rescue of the gonadal sex reversal when *Foxl2* was simultaneously ablated, thereby suggesting that testicular differentiation involves FGFR2c-mediated repression of the FOXL2-driven promotion of ovarian differentiation [90]. Moreover, antagonistic interactions between *Sox9*/*Rspo1* [91] and *Sox9*/*β-catenin* [92] have been reported in mice, with testicular differentiation observed in both *Sox9*/*Rspo1* and *Sox9*/*β-catenin* double knockouts. These findings confirm that male and female gonadal sex differentiation are both induced by distinct genetic pathways and that repression of the opposite pathway is essential to ensure normal sex-specific gonadal development. 

In human fetal gonads, *WNT4* expression does not appear to be sex-specific or show temporal fluctuations, whereas *RSPO1* expression is ovary-specific [93,94]. Nevertheless, both *WNT4* and *RSPO1* are important in the promotion of ovarian development in humans based on evidence from patients with mutations in these genes. Loss-of-function mutations in *WNT4* have been identified in 46,XX individuals that are virilized and lack Müllerian structures [95,96], while loss-of-function mutations in *RSPO1* leads to 46,XX DSD with complete sex-reversal [97], or 46,XX ovotesticular DSD, although no information about fertility was reported in these patients [98]. Conversely, duplication of chromosome 1p31-p35 (which contains both *WNT4* and *RSPO1*) has been reported to cause 46,XY male-to-female sex reversal, suggesting that genes important in human gonadal sex differentiation may be dosage-sensitive [99]. However, neither *WNT4* nor *RSPO1* were duplicated in another more recent case of male-to-female sex reversal with partial duplication of 1p, thereby suggesting that other genes in this region may contribute or indeed be the cause of the observed gonadal phenotype [100].

Interestingly, the WNT signaling antagonist ZNRF3, which is also a direct target of RSPO1-mediated inhibition, was recently shown to be required for testicular differentiation in mice, with XY mice lacking *Znrf3* exhibiting complete or partial gonadal sex reversal [101]. In accordance, three human *ZNRF3* variants were identified in rare cases of 46,XY female DSD, thereby identifying a testis-determining function for ZNRF3 in humans and suggesting an antagonistic relationship between ZNRF3 and RSPO1 also in human gonadal sex differentiation [101]. In the five patients with *ZNRF3* variants and 46,XY DSD varying degrees of gonadal dysgenesis was reported, but no information about fertility was included [101]. 

Following initiation of the female promoting pathway by WNT4/RSPO1/β-catenin signaling, granulosa cell fate and ovarian development is enforced by expression of the transcription factor FOXL2 in mice [102,103,104,105,106]. It is likely that the WNT4/RSPO1/β-catenin signaling pathway contributes to the upregulation of FOXL2 in granulosa cells, which in human fetal ovaries are expressed in a sub-population of the somatic cells from around 10 weeks post-conception [107] although the mechanism through which this may be mediated is not understood. In humans, an autosomal dominant mutation in FOXL2 has been associated with premature ovarian failure, but not sex reversal [108,109], suggesting that also in humans FOXL2 is not essential for the initial establishment of the granulosa cell population.

## 4. Maintenance of Sex-Specific Somatic Cell Lineages

Importantly, the carefully regulated balance between the promotion of testicular or ovarian differentiation and simultaneous suppression of the opposite pathway during embryonic and fetal development is not the final sex-fate decision. Genetic studies in mice have shown that sex-specific gonadal fates must be actively maintained in adulthood, emphasized by the continued requirement to maintain Sertoli or granulosa somatic cell fate.

### 4.1. FOXL2: Maintenance of Female Fate

Studies in *Foxl2* null mice have demonstrated numerous effects on ovarian function [104,106], including upregulation of *Sox9* after birth, which suggests a continuous requirement for FOXL2 to maintain granulosa cell identity throughout development and maturation [103]. In accordance, conditional deletion of *Foxl2* in adult ovarian follicles of mice resulted in upregulation of testis-specific genes, including *Sox9* thereby demonstrating reprogramming of granulosa cells into Sertoli cell-like cells [105]. Additionally, steroid hormone production was altered in females with ablated *Foxl2*, with testosterone levels comparable to those of normal XY littermates, thereby suggesting reprogramming of theca cells towards a Leydig cell-like fate [105]. 

Additional detailed analyses demonstrated that *Foxl2* interacts with estrogen receptors through TESCO, the gonad-specific enhancer of SOX9, thereby suppressing *Sox9* expression and reprogramming of granulosa cells in the adult mouse ovary [105,110]. Conversely, transgenic gonadal expression of *Foxl2* in XY mice resulted in ovotestis-like gonads at 13.5 dpc with disrupted tubular structures and reduced AMH expression [111]. In accordance, male-to-female sex reversal was observed in XY *Fgfr2c^−/−^* gonads in which upregulation of *Foxl2* was reported [112]. 

Consistent with the role of RSPO1/WNT4/β-catenin and FOXL2 in the promotion and maintenance of ovarian fate, the combined loss of *Foxl2* and *Wnt4*, or *Foxl2* and *Rspo1* results in female-to-male sex reversal that occurs earlier and with a more severe phenotype than sex reversal resulting from loss of *Foxl2*, *Wnt4*, or *Rspo1* alone [111,113]. Together, these studies indicate that FOXL2 is required for the maintenance of the granulosa cell lineage in ovaries and suppression of the testicular pathway by preventing trans-differentiation of granulosa cells into Sertoli cells, thereby emphasizing that maintenance of the ovarian phenotype continues throughout life.

### 4.2. DMRT1: Maintenance of Male Fate

Consistent with the concept of continued maintenance of sex-specific gonadal fate, studies in mice have identified DMRT1 as an essential factor responsible for maintaining the Sertoli cell lineage and testicular fate. *Dmrt1^−/−^* XY mice are born with testes [114], but these undergo abnormal differentiation during postnatal development with loss of *Sox9* expression and upregulation of *Foxl2* [115]. In accordance, conditional loss of *Dmrt1* in adult testis results in upregulation of FOXL2 expression, reprogramming of Sertoli cells into granulosa cells, and presence of theca-like cells and germ cells that appeared feminized [115]. This suggests that in mice *Dmrt1* is essential to maintain testis determination and antagonize female promoting factors (mainly FOXL2) throughout life. Conversely, induced expression of *Dmrt1* in XX gonads was recently reported to reprogram sex-specific gonadal differentiation and promote testicular development in mice [10,116]. 

Ectopic expression of *Dmrt1* in the ovary resulted in downregulation of the female sex-maintenance gene *Foxl2*, reprogramming of granulosa cells to Sertoli-like cells, and formation of structures resembling male seminiferous cords [10]. In accordance, an independent study found that overexpression of *Dmrt1* in XX gonads was indeed sufficient to promote testicular differentiation [116]. Additionally, these transgenic XX gonads had typical testicular vasculature, reduced expression of *Foxl2*, and induction of *Sox9* expression. Also, fetal Leydig-like cells and non-meiotic germ cells were found in the transgenic XX gonads, but in this model formation of seminiferous cords was not observed [116]. Together these results suggest that ectopic expression of *Dmrt1* in ovaries is sufficient to promote testicular differentiation in mice even though it is dispensable for the initial establishment of Sertoli cell fate during fetal life. 

In humans, loss of *DMRT1* has been reported in relation to deletions of chromosome 9p24, which results in varying degrees of 46,XY gonadal dysgenesis [117,118,119,120], suggesting that DMRT1 may contribute to the maintenance of male fate in human supporting cells. Whilst it is likely that cases of 46,XY DSD in individuals with deletions of chromosome 9p24 are due to loss of *DMRT1*, it cannot be excluded that other genes in this region contribute, or indeed cause, the observed gonadal phenotypes [121,122,123]. In accordance with the former hypothesis, knockdown of *DMRT1* expression in a human fetal testis ex vivo model induced focal testicular dysgenesis and expression of FOXL2 in a small sub-population of supporting cells in which SOX9 expression was lost [124].

### 4.3. DHX37: A Novel Participant of Gonadal Development and Maintenance

Variants of the DEAH-box helicase 37 (*DHX37*) gene have recently been identified as an important cause of 46,XY GD, especially for embryonic testicular regression syndrome (ETRS). *DHX37* is an RNA-helicase which has a role of DHX37 in ribosome biogenesis [125] and is expressed in the somatic cell lineage of the mouse and human (7–12 weeks of gestation) gonad early during testis determination and development [126]. Co-expression with *Sox9* in a proportion of cells indicates the presence of DHX37 in Sertoli cells [126], whilst it is also expressed in Leydig cell cytoplasm and in germ cells at different stages of maturation (27 and 33 weeks of gestation) and, in adult human testes, the protein is mainly localized in spermatogonia [126,127].

Four heterozygous missense rare variants classified as pathogenic or likely pathogenic in *DHX37* have been reported in 11 patients from five families and in six sporadic cases with gonadal dysgenesis and also ETRS [127]. Seven different missense heterozygous *DHX37* variants were also identified in 13 children from another cohort with similar phenotypes [126]. All the identified variants are clustered in two highly conserved functional domains and were specifically associated with gonadal dysgenesis and ETRS in both cohorts. Segregation analysis of the *DHX37* variants displayed a sex-limited autosomal dominant pattern, mostly de novo or maternally inherited and paternally inherited in one family. The p.Arg308Gln, p.Arg674Trp, and p.Thr304Met were the most common *DHX37* variants identified. In both cohorts, the frequency of *DHX37* deleterious variants were similar to the *NR5A1* variants, accounting for 14% and 11% of the 46,XY GD patients, respectively [126,127]. In a third cohort of 46,XY DSD adult women, *DHX37* variants accounted for 15.4% of the partially virilized individuals [128]. 

The fact that the same variant could be observed in individuals with gonadal dysgenesis and others with ETRS reinforces that these phenotypes are part of the same clinical spectrum and indicates that this gene is important not only for gonadal determination, but also its maintenance. 

Despite the fact that the precise mechanism by which *DHX37* variants lead to GD remains to be elucidated, the present genetic evidence highlights ribosomopathies as a novel mechanism for DSD. 

Regarding fertility issues, curiously, in one of the reported families, there is one fertile father who harbored the recurrent p.Arg308Gln *DHX37* deleterious variant, resembling *NR5A1* variants [127].

**Figure 1:**
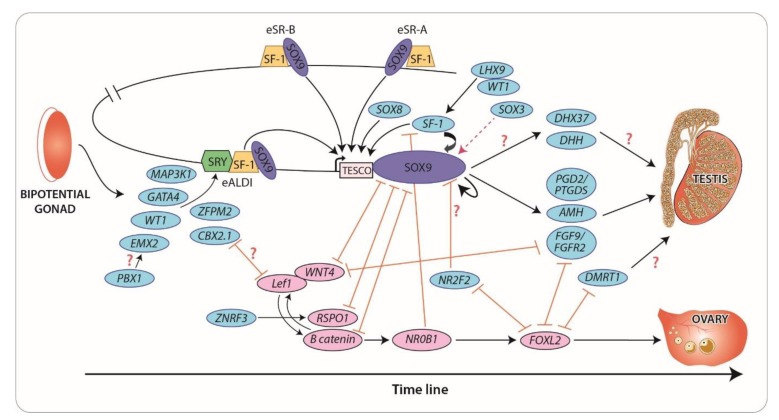
Timeline of genes involved in gonadal development. Genes shown are known to play a role in sex-specific gonadal development in human and mice. Testis-related genes (blue) and ovary-related genes (pink) represent a regulatory pathway which leads to Sertoli and granulosa cell development, respectively. The orange arrows represent an antagonistic action. Interactions that are postulated but unproven are indicated with (?). The ectopic gonadal expression of Sox3 (represented by red dotted line) induces testis differentiation by upregulating Sox9 in a similar way to Sry.

Current understanding of the timeline and genetic interactions that are required for mammalian sex determination and differentiation are summarized in Figure 1.

## 5. Impact of DSD on Fertility and Options for Fertility Preservation

The impact of DSD on fertility will depend on several factors. The key determinant of fertility potential will be gonadal development and function, and ultimately whether the individual has ovarian tissue with viable oocytes, or a testis capable of producing functional sperm. Whilst fertility preservation options are well established for many patient groups such as those receiving gonadotoxic treatment for cancer [129], there are a number of important additional considerations for those with DSD that must be evaluated on an individual patient basis (Table 1).

### 5.1. Sex Chromosome DSD

The relationship between DSD and impaired fertility is well established, especially for the two most common sex chromosome DSDs. For individuals with Turner syndrome (45,X) and Klinefelter syndrome (47,XXY), primary gonadal failure is common. For both disorders, pubertal delay with hypergonadotrophic hypogonadism is frequent, resulting in sex steroid deficiency and infertility. 

#### 5.1.1. Turner Syndrome

Turner syndrome (TS) affects 1:2000 girls and the majority (95–98%) of individuals with TS are infertile [130]. For most individuals with TS, primary ovarian failure occurs during prepuberty and spontaneous menarche has been reported to occur in only 20% (95/480) of cases in one large cohort of patients [131]. Spontaneous pregnancy is rare in individuals with TS, occurring in only 2–5%, with an increased rate of fetal anomalies and pregnancy loss also reported [132]. Infertility results from an accelerated loss of oocytes which begins in fetal life and progresses during postnatal life [130]. A complete 45,X karyotype has been reported to be predictive of ovaries without presence of follicles, whereas a mosaic karyotype and spontaneous menarche increase the likelihood of follicles being present [133]. In a cohort of 15 individuals (aged 5–22 years) with TS, follicles were identified in 9 (60%) and in the majority of these cases (7/9) the follicle counts were within the 95% confidence interval for the normal population. All were mosaic, except for a 5-year-old girl with a 45,X karyotype. However, despite the presence of follicles in the ovaries of the majority of those TS cases, the morphology of the follicles was frequently abnormal, suggesting that the functional capacity of these oocytes may be impaired [133].

For post-pubertal patients it may be possible to perform ovarian stimulation and oocyte retrieval; however, this is entirely dependent on the presence follicles from which oocytes can be retrieved. For the majority of individuals with TS it is not possible to preserve fertility and in these cases counseling regarding fertility should be offered and egg/embryo donation or adoption considered where appropriate.

Whilst ovarian tissue cryopreservation (OTC) has been shown to be a successful strategy for fertility preservation in young females at risk of infertility due to planned gonadotoxic therapies, it is not clear whether this is a suitable option for individuals with Turner syndrome. Although spontaneous menarche, mosaic karyotype, detectable AMH, and normal follicle stimulating hormone (FSH) may offer some prediction about the presence of follicles, they do not predict the functional capacity of the oocytes within the cryopreserved tissue [133]. Given the abnormal morphology of the follicles, fertility restoration by re-transplantation of cryopreserved ovarian tissue is unlikely to be successful and therefore subsequent use of cryopreserved ovarian tissue would likely require ex vivo oocyte maturation and artificial reproductive technologies. To date, there have been no reports of restoration of fertility using cryopreserved ovarian tissues from individuals with TS. 

#### 5.1.2. Klinefelter Syndrome

Klinefelter syndrome (KS) is common, occurring in 1:600 live births. KS is associated with hypogonadism and infertility with fewer than half of adult men reported to have spermatozoa present in the ejaculate, although residual foci with spermatogenesis may be present in some individuals with apparent azoospermia [134]. The testicular phenotype of KS involves a progressive loss of spermatogonial stem cells (SSC) beginning in prepuberty, with testicular fibrosis occurring in (peri)pubertal and adult patients [135]. A meta-analysis of the presence of spermatogonia in individuals with KS demonstrated spermatogonia in the testes of all fetal/infantile samples, 83% of those obtained from prepubertal patients, and in 40–50% of adolescent/adult individuals [20].

Options for fertility preservation in individuals with KS are dependent on the age of the patient. For post-pubertal patients, the primary goal is to obtain viable spermatozoa that can be stored for future use in artificial reproductive technologies. This involves surgical intervention to retrieve sperm using testicular sperm extraction (TESE). According to a recent systematic review and meta-analysis, TESE has been shown to result in successful retrieval of sperm in ~40% of cases [134]. Interestingly, clinical and biochemical parameters, such as age, testis volume, and hormone status at baseline, were not predictive of successful sperm retrieval [134]. A total of 29 studies in this meta-analysis reported fertility outcomes using sperm retrieved by TESE with pregnancy and live-birth rate of 43% [134].

For prepubertal boys, it is not possible to obtain sperm and therefore the only option is to cryopreserve and store testicular tissue with the aim of preserving SSCs that can be used to generate sperm for future use in artificial reproductive technologies. Whilst testicular tissues are increasingly being stored from prepubertal boys due to receive gonadotoxic therapies, there are no proven clinical methods to generate sperm from these tissues [136]. Furthermore, the underlying testicular and germ cell abnormalities in individuals with KS may result in additional challenges for the use of this approach for fertility preservation. Therefore, this approach has recently been called into question [135] and testicular tissue cryopreservation in prepubertal patients should only be considered as part of an ethically approved research study.

**Figure 2:**
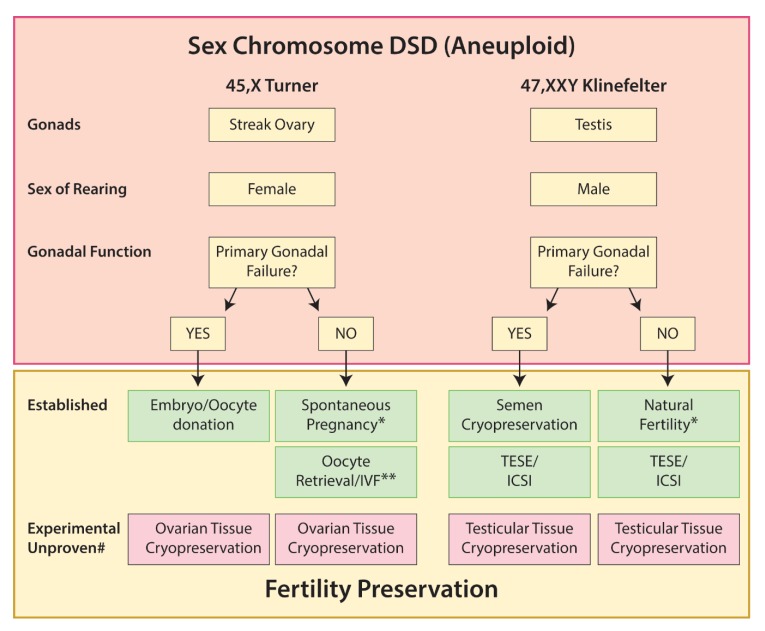
Options for fertility preservation in sex chromosome DSD (aneuploid). * Mostly mosaics. ** Postpubertal. # Potential for gonadal tissue cryopreservation based on published literature indicating presence of germ cells +/− tissue cryopreservation; however, there are no reports of successful restoration of fertility and this should be considered within the context of an ethically approved research study.

Options for fertility preservation in 45,X and 47,XXY are summarized in Figure 2.

#### 5.1.3. 45,X/46,XY

The phenotype of individuals with 45,X/46,XY depends on the proportion and distribution of each sex chromosome complement (45,X or 46,XY) within the body tissues. As a result, the appearance of 45,X/46,XY can range from that of 45,X (Turner syndrome) female to a phenotypic male. 45,X/46,XY is the most common cause of mixed gonadal dysgenesis in which the gonads are a combination of a streak gonad and a contralateral testis [137]. For those individuals raised as male, spontaneous puberty usually occurs, although testosterone may be required to complete puberty [137]. Fertility in individuals with 45,X/46,XY is dependent on the potential for sperm production in the testis, although the testis tissue is frequently dysgenetic. Whilst it is generally considered that individuals with 45,X/46,XY are infertile, there are isolated case reports of fertility in both males and females. A single case of successful sperm extraction was reported in a patient with azoospermia [138]. This individual had a 50:50 split in mosaicism and 96% of the retrieved sperm were aneuploid. Sperm were cryopreserved from this patient, but no information about the functionality of the sperm was reported [138]. A recent study in a relatively large (*n* = 63) cohort of males with 45,X/46,XY mosaicism showed that the majority (79%) entered puberty spontaneously, but 39% of all individuals in the cohort required testosterone supplementation at some stage during follow-up [139]. Whilst the gonadal phenotype and histology was highly variable, the majority of patients had dysgenetic testes. In the majority of postpubertal patients no evidence of spermatogenesis was found, although 25% had focal areas with spermatogenesis. Seventeen patients had semen analyzed and live spermatozoa were present in 17.6% (3/17), albeit with low total number of sperm [139]. Spontaneous pregnancies have been reported in females with mosaicism [140]. Remarkably, a spontaneous pregnancy has been reported in a female with a predominantly 46,XY karyotype, including in the gonad (95% 46,XY) [141]. A recent study of 44 individuals with Turner syndrome and presence of Y chromosome material found spontaneous menarche in 2/26 (7.6%) of those with a 45,X/46,XY karyotype, although the potential for fertility in these individuals was not reported [142].

For males the options are similar to those described for KS. However, an additional consideration in all individuals those with a 45,X/46,XY karyotype is the increased risk of gonadal malignancy that may necessitate gonadectomy [22].

#### 5.1.4. 45,XX/46,XY

45,XX/46,XY DSD may arise as a result of mosaicism or true chimerism [21,143]. Phenotypes range from typical male to typical female. Gonads in individuals with 46,XX/46,XY DSD may include testicular and ovarian tissues. In phenotypic females, spontaneous pregnancies have been reported [144]. For males, azoospermia is considered almost universal, although there are reports of successful TESE and subsequent pregnancies using the cryopreserved sperm [145,146] and a case of an azoospermic male whose sperm count became normal after Mullerian structures had been removed [143]. These cases indicated that fertility preservation may be possible in some 46,XX/46,XY individuals using semen cryopreservation or TESE.

**Figure 3:**
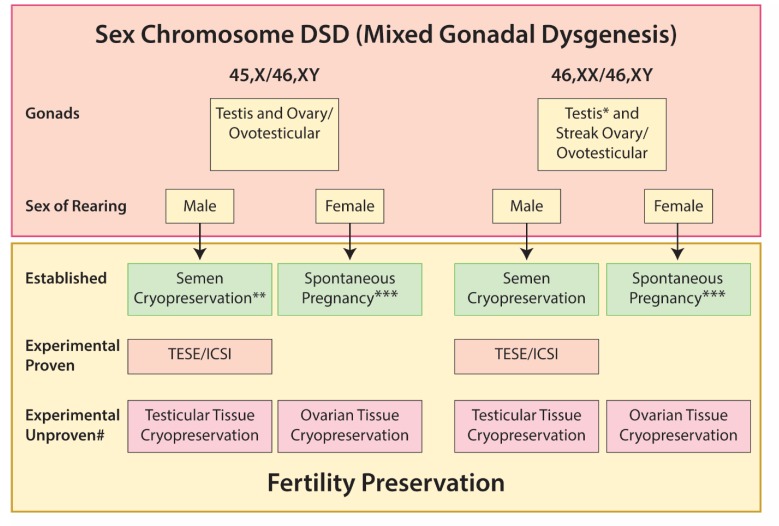
Options for fertility preservation in sex chromosome DSD (mixed gonadal dysgenesis). * Usually dysgenetic. ** May be possible for IVF. *** Rare case reports. # Potential for gonadal tissue cryopreservation based on published literature indicating presence of germ cells +/− tissue cryopreservation; however, there are no reports of successful restoration of fertility and this should be considered within the context of an ethically approved research study.

Options for fertility preservation in 46,XX/46,XY and 46X/46,XY DSD are summarized in Figure 3.

### 5.2. 46,XX DSD

#### 5.2.1. Disorders of Androgen Production or Action

The majority of 46,XX DSD are due to excess androgen production. Most commonly this results from CAH due to 21-hydroxylase deficiency. The primary pathology is due to overproduction of adrenal androgens, as opposed to a failure of normal ovarian development. As a result, XX individuals with 21-hydroxylase CAH may be fertile [132]. However, women with CAH are reported to have lower pregnancy rates when compared to age-matched controls [147]. Live-birth rates of 33–50% have been reported in those with ‘simple virilizing’ forms of CAH [24]. However, the likelihood of fertility is significantly lower in those with severe ‘salt-wasting’ forms of CAH in whom live-birth rates of 0–10% have been reported [24]. In non-classical forms of CAH due to partial enzyme deficiencies, live-birth rates are higher (63–90%) than classical CAH and are similar to age-matched controls [24]. In more rare forms of 46,XX CAH that may also affect gonadal steroid production, fertility is rarely described, other than in few case reports of a spontaneous pregnancy, e.g., in an individual with 3β-hydroxysteroid dehydrogenase deficiency [148], and successful in vitro fertilization (IVF) in individuals with 17α-hydroxylase deficiency [149,150,151], and another with congenital lipoid adrenal hyperplasia [152]. In CAH, infertility may occur as a result of anovulation, menstrual irregularities, thickening of cervical mucus, and anatomical factors [132]. In addition, there are important psychosocial factors that impact on fertility in individuals with CAH, which may include the lack of a steady relationship or a reduction in those trying for a pregnancy compared to the general population [147]. Overall, the data indicate that the likelihood of fertility is associated with disease severity and disease control. Therefore, the mainstay of treatment involves optimizing androgen levels with appropriate steroid therapy [132].

#### 5.2.2. 46,XX Testicular DSD

46,XX testicular DSD is characterized by the presence of testicular tissue despite the absence of the Y chromosome. The majority of these individuals have a translocation of the *SRY* gene, accounting for 80% of those with non-syndromic 46,XX testicular DSD with phenotypically male external genitalia [153]. Despite the presence of the *SRY* gene, impaired spermatogenesis in these individuals may be due to the lack of other Y chromosome genes that are known to be important for fertility, such as the azoospermia (AZF) region [154]. The phenotype in individuals with 46,XX testicular DSD may resemble KS and infertility with azoospermia is reported to be universal in this population [29,137]. 

**Figure 4:**
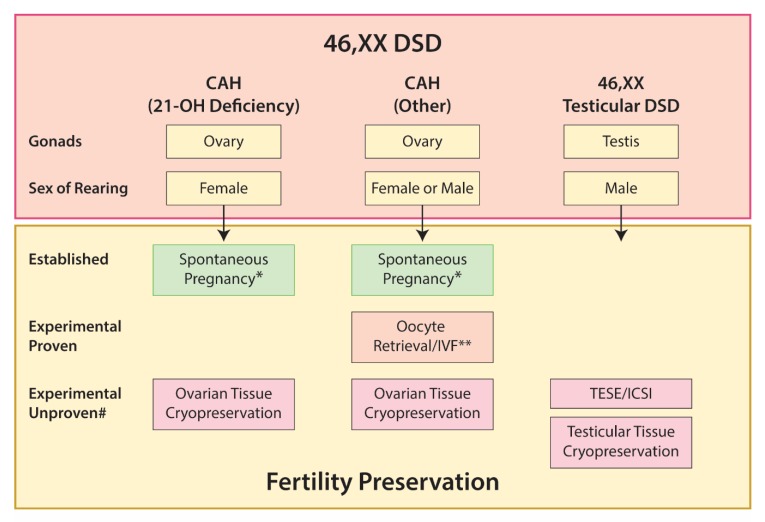
Options for fertility preservation in 46,XX DSD. * Optimise therapy for CAH. ** Postpubertal. # Potential for gonadal tissue cryopreservation based on published literature indicating presence of germ cells +/− tissue cryopreservation; however, there are no reports of successful restoration of fertility and this should be considered within the context of an ethically approved research study.

Options for fertility preservation in 46,XX DSD are summarized in Figure 4.

### 5.3. 46,XY DSD

#### 5.3.1. Gonadal Dysgenesis

Complete gonadal dysgenesis in 46,XY individuals results from mutations in key testis-determining genes in a phenotypic female with internal Mullerian structures and bilateral streak gonads. The lack of either testicular or ovarian tissue means that it is not possible to obtain spermatozoa or oocytes from these individuals and therefore infertility is universal. However, the presence of Mullerian structures means that pregnancy may be possible using donor eggs or embryos and IVF, as illustrated by a recent report of successful pregnancies in two sisters, with a healthy live birth in one and an ongoing pregnancy in the other [155]. 

Individuals with 46,XY partial gonadal dysgenesis (PGD) present with variable genital ambiguity and varying degrees of testicular dysgenesis or streak gonads [156]. Whilst severe oligozoospermia has been reported in a long-term follow-up study of males with PGD [156], for phenotypic males with mild abnormalities of gonadal development or external genitalia (e.g., hypospadias), fertility may be possible.

**Figure 5:**
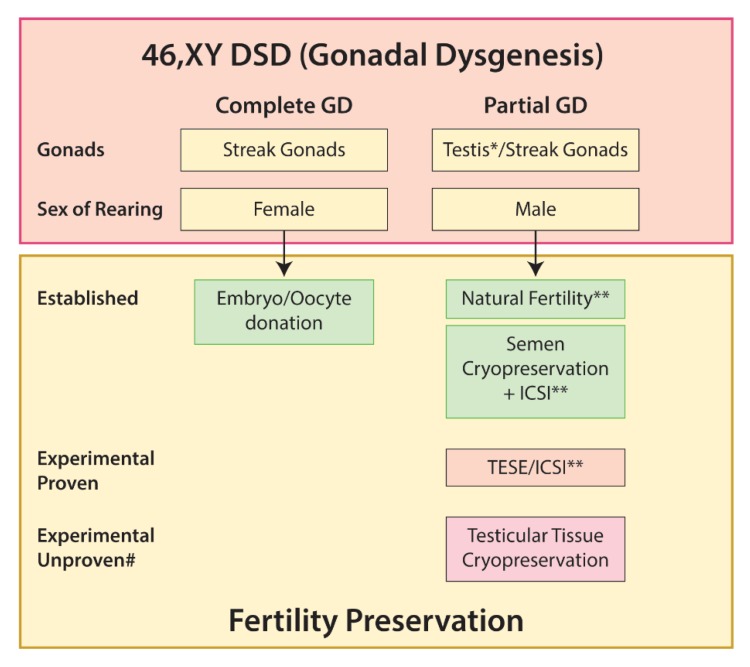
Options for fertility preservation in 46,XY DSD with gonadal dysgenesis. GD—gonadal dysgenesis. * Often dysgenetic. ** May be possible for milder phenotypes. # Potential for gonadal tissue cryopreservation based on published literature indicating presence of germ cells +/− tissue cryopreservation; however, there are no reports of successful restoration of fertility and this should be considered within the context of an ethically approved research study.

Options for fertility preservation in 46,XY gonadal dysgenesis DSD are summarized in Figure 5.

#### 5.3.2. Disorders of Androgen Production

46,XY males with 21-hydoxylase CAH are reported to have reduced fertility and semen parameters [24,157]. Fertility appears to be largely dependent on good control of excess androgens and avoidance of over-treatment with steroids [24]. Testicular adrenal rest tumors (TART) can occur in individuals with CAH. Whilst failure to suppress excess adrenal androgen production can promote their development, it is also recognized that TARTs can occur in some patients who are well-controlled [24]. The presence of bilateral TARTs with pressure and destruction of the testicular tissue may also result in primary gonadal failure [24]. Hypogonadotrophic hypogonadism may occur in males with CAH as a consequence of the elevated circulating androgens. Similar to females with CAH, optimal control of adrenal androgen production represents the main approach to preserving fertility potential. Recovery of a normal sperm count in an azoospermic man following treatment with a long-acting glucocorticoid was recently reported [158], although the benefits and potential risks of this form of therapy remain unclear [24].

For other disorders of steroidogenic enzymes in 46,XY individuals, mutations may affect gonadal steroidogenesis. These disorders result in varying degrees of sex-reversal often with delayed puberty and infertility [137]. Fertility and paternity are rarely reported in these patients, although there is some evidence to suggest that non-classic forms in which there is partial enzyme activity, and the individual is raised as male, may have normal puberty and fertility [137,159].

Mutations in the LH receptor resulting in reduced testicular androgen production will result in similar effects. For those with complete forms, phenotypic appearance is often female and therefore sex-of-rearing is usually female. In such cases, pubertal induction with estrogen is required and gonadectomy is often performed due to increased risk of gonadal malignancy. Whilst individuals with Leydig cell hypoplasia are considered to be azoospermic, a case-report has described an individual with an inactivating mutation of the LH receptor from whom sperm were successfully obtained by TESE after treatment with hCG [160]. The cryopreserved sperm were used for intra-cytoplasmic sperm injection (ICSI). resulting in a successful pregnancy and live birth [160]. Mutations in 5-α-reductase gene results in failure to convert testosterone to DHT. The phenotype is variable and can range from ambiguous genitalia to typical female appearance. Gender reassignment from female to male may occur as a result of virilization at puberty [137]. Spontaneous fertility is believed to be rare in individuals with 5-α-reductase deficiency as a result of frequent abnormalities in spermatogenesis and semen parameters, including increased semen viscosity and reduced semen volume [26]. However, successful pregnancies and births have been reported following intra-uterine insemination (IUI) or ICSI using semen samples obtained from patients [26,161].

For fertility preservation in young males with disorders of androgen production, semen cryopreservation may be considered, whilst for those with azoospermia, TESE may be an option to obtain sperm for assisted reproductive technologies (ART) [24,137], although the success rates based on individual disorders or specific mutations are often unknown.

**Figure 6:**
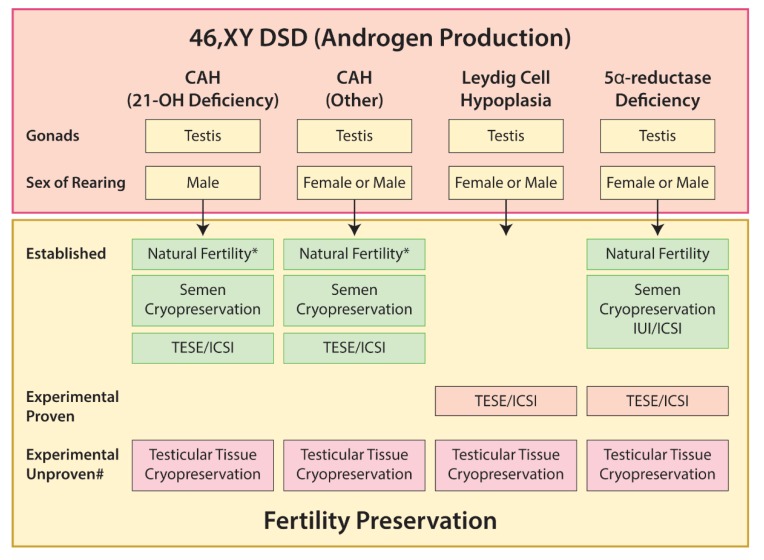
Options for fertility preservation in 46,XY DSD associated with impaired androgen production. * Optimise therapy for CAH. # Potential for gonadal tissue cryopreservation based on published literature indicating presence of germ cells +/− tissue cryopreservation; however, there are no reports of successful restoration of fertility and this should be considered within the context of an ethically approved research study.

Options for fertility preservation in 46,XY DSD due to impaired androgen production are summarized in Figure 6.

#### 5.3.3. Disorders of Androgen Action

##### Androgen Insensitivity Syndrome

Individuals with CAIS are born with a female phenotype and are usually not diagnosed until childhood when they present with an inguinal hernia, which includes a gonad, or later with primary amenorrhoea. Mullerian structures are not present, precluding patients with CAIS from carrying a pregnancy. The gonads are composed of testicular tissue, although the histology is highly abnormal with a rapid decline in germ cell number beginning in infancy [28]. Spermatogonia are present in the gonads of ~30% of adults with CAIS but the majority of tubules are Sertoli-cell-only [87]. Presence of spermatozoa has not been described in the testis of an individual with CAIS and therefore the potential for fertility (e.g., using ICSI) is not possible.

The phenotype of PAIS is highly variable, ranging from ambiguous genitalia in severe cases to cryptorchidism, hypospadias, and infertility in classical cases. Whilst subfertility is generally considered universal for individuals with PAIS raised as male, spontaneous natural fertility was reported in one case [162] and following stimulation with high-dose testosterone and subsequent ICSI [163]. In other cases of subfertility, IVF may also be possible [137].

For 46,XY females with disorders of androgen action, there are no options for fertility preservation due to the lack of viable gametes and Mullerian structures. For the majority of males, sperm retrieval is not an option as spermatogenesis is not present in these testes [28,137]. Therefore, future strategies for fertility preservation in these individuals are likely to depend on the presence of spermatogonia within the gonad. Such an approach would require testicular tissue cryopreservation and subsequent development of spermatozoa from the stored tissue using in vitro and transplantation techniques, which are being developed as a strategy for fertility preservation in individuals facing gonadotoxic therapies [136]. However, additional considerations for these individuals include underlying abnormality of the testis that may prevent development of spermatozoa [28,164] and the risk of gonadal malignancy for those with AIS [28,165].

**Figure 7:**
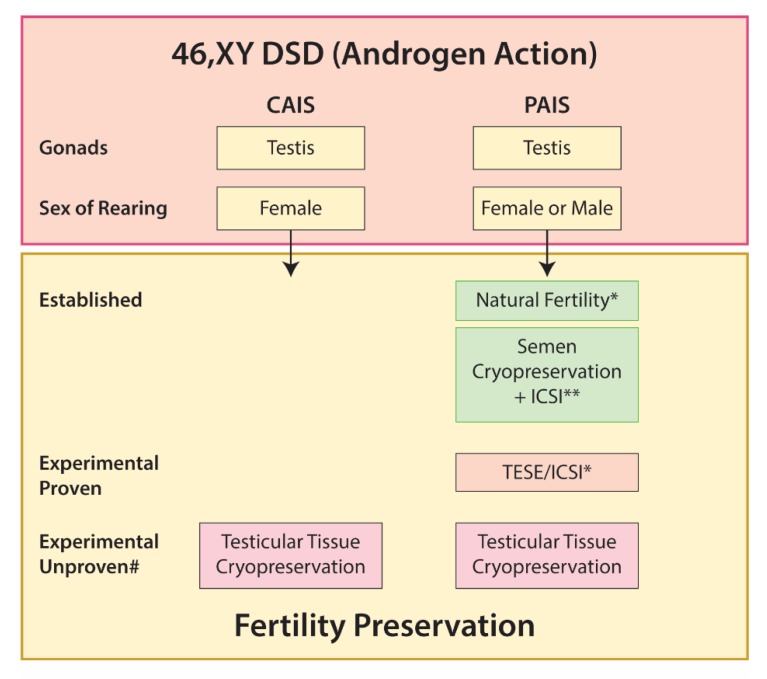
Options for fertility preservation in 46,XY DSD associated with impaired androgen action. * Single case report. ** Single case report (high-dose testosterone). # Potential for gonadal tissue cryopreservation based on published literature indicating presence of germ cells +/− tissue cryopreservation; however, there are no reports of successful restoration of fertility and this should be considered within the context of an ethically approved research study.

Options for fertility preservation in 46,XY DSD due to impaired androgen action are summarized in Figure 7.

### 5.4. Ovotesticular DSD

Ovotesticular DSD represents a unique group of DSD in which there is presence of ovarian and testicular tissues in individuals with a 46,XX, 46,XY, or 46,XX/46,XY karyotype. The underlying genetic causes and associated phenotypes are variable, as are the internal and external genitalia. As a result, the potential for fertility is largely dependent on gonadal pathology, reproductive tissues, and whether the individual is raised as male or female. The ovarian component of the gonad tends to have a relatively normal histology, whereas the testicular component is frequently dysgenetic, with limited germ cells and rare spermatozoa [137]. Whilst spontaneous pregnancies have been reported in 11 females with ovotesticular DSD [166], only one successful paternity has been described in a 46,XX/46,XY male with ovotesticular DSD, after TESE and ICSI [145].

For phenotypic males, fertility preservation options are similar to those described for individuals with 46,XY GD DSD. For phenotypic females with ovotesticular DSD who are unable to achieve a natural pregnancy, oocyte retrieval for ART may be possible, although this will also depend on the presence and anatomy of Mullerian structures and the potential for the individual to carry a pregnancy to term. Recent advances in uterine transplant have increased the possibilities for individuals with DSD who lack Mullerian structures. Whilst this has primarily been applied to a small number of individuals with uterine factor infertility e.g., Mayer–Rokitansky–Küster–Hauser syndrome [167], in the future this may be applied to other cases in which Mullerian structures are not present.

## 6. Fertility Preservation in Prepubertal Individuals with DSD—Ovarian or Testicular Tissue Cryopreservation

Over recent years there has been a growing interest in fertility preservation in prepubertal patients from whom it is not possible to obtain sperm or eggs. This has largely been applied to children due to receive gonadotoxic treatment for cancer [168]. For these patients, the only option available is to cryopreserve and store gonadal tissue with the aim of preserving germ cells that can be used to generate gametes for ART or for re-transplantation to restore fertility [169]. Whilst ovarian cryopreservation and re-transplantation has successfully restored fertility in females, there are no proven clinical methods to restore fertility using prepubertal testis tissues [136].

Cryopreservation of gonadal tissues from young individuals with DSD has recently been reported in 10 patients undergoing gonadectomy for a variety of DSD diagnoses [170]. This study demonstrated the presence of germ cells in 5/10 patients and 4 of these elected for tissue cryopreservation. This included four females (two individuals with mixed gonadal dysgenesis, one with PAIS and 1 with OT DSD) and one male (with partial gonadal dysgenesis). Of the 5 females with no germ cells present, three had mixed gonadal dysgenesis (Turner Syndrome with Y-chromosome material), whilst the other two had complete gonadal dysgenesis and tissue was not cryopreserved in these individuals [170]. Important considerations for future development of such an approach will be to consider the potential for obtaining and differentiating viable germ cells, optimal timing of gonadectomy in relation to the risk of gonadal malignancy balanced against the likelihood of successful restoration of fertility using the tissue. Furthermore, the application of such an approach to patients in whom gonadectomy is not clinically indicated must be carefully considered from an ethical perspective with consideration of the clinical risks and benefits of surgery. Whilst gonadal tissue cryopreservation may ultimately lead to new strategies for fertility preservation in DSD, this approach should currently be considered only in the context of an ethically approved research study.

## 7. Conclusions

Significant progress has been made in understanding the mechanisms that underlie the development of DSD in humans. The complexity of gene interactions that drive development of the bipotential gonad to either a testis or an ovary are increasingly recognized. In many cases, this involves a delicate balance between ‘promoting’, ‘inhibiting’ and ‘antagonistic’ signaling pathways, which results in a wide variation in clinical phenotype for individuals with DSD. Despite the fact that fertility is often impaired in DSD, established options for fertility preservation are limited and there are important ethical and practical considerations for the development of future fertility therapies in this population. Future research should focus on developing safe and effective strategies that may preserve fertility potential in individuals with DSD.

## Figures and Tables

**Table 1:** Key considerations for fertility and fertility preservation in DSD.

Consideration	Management	Implications for Fertility
Malignancy risk	Gonadectomy	- Natural fertility not possible - Gametes may be obtained at surgery - Gonadal tissue may be obtained at surgery
	Conservative	- Natural fertility may be possible - gametes or gonadal tissue may be obtained in adulthood
Progressive germ cell loss	Fertility preservation	- Gamete/tissue retrieval only possibleprior to loss
Gonads/gametes incongruent to sex of rearing	Fertility counseling	- Gametes may be obtained and stored - Gonadal tissue may be cryopreserved
Transmission of genetic abnormality to subsequent generations	Genetic/fertility counseling	- Specific diagnosis/variant may influence pregnancy outcomes - Potential risk of recurrence in offspring
Presence/absence of Mullerian structures	Radiology/surgical assessment	- Potential for carrying a fetus to term may be limited - Absence of birth canal will prevent vaginal delivery
